# Supplementary material for: Plasma proteins are integral to gene-regulatory networks acting within and across blood cells, the arterial wall and major metabolic organs
Source: medRxiv. 2025 Jan 25:2025.01.22.25320723. Preprint. [Version 1] doi: 10.1101/2025.01.22.25320723 (PMC11839005; doi:10.1101/2025.01.22.25320723)
Supplement: Supplement 2 [file NIHPP2025.01.22.25320723v1-supplement-2.pdf]

medRxiv preprint doi: https://doi.org/10.1101/2025.01.22.25320723; this version posted January 25, 2025. The copyright holder for this preprint (which was not certified by peer review) is the author/funder, who has granted medRxiv a license to display the preprint in perpetuity.
It is made available under a CC-BY 4.0 International license .

SUPPLEMENTARY MATERIAL

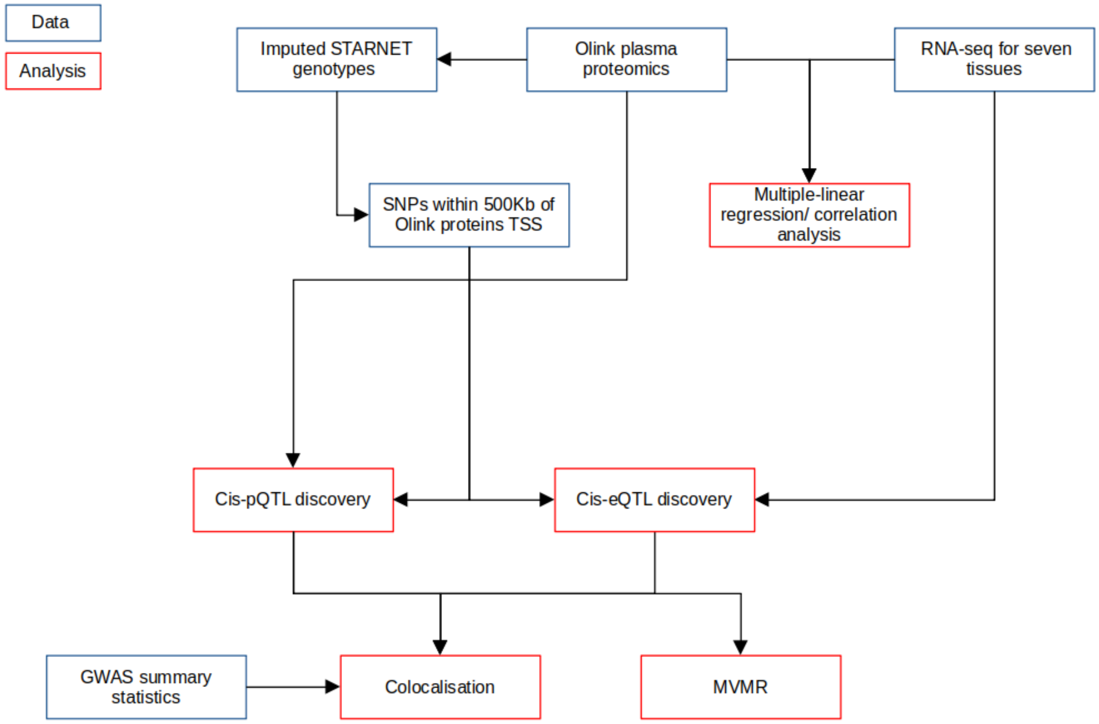

Figure S1: Analysis work-flow diagram. Blue boxes represent data inputs and red boxes indicate analyses. Arrows indicate flow of information.

medRxiv preprint doi: https://doi.org/10.1101/2025.01.22.25320723; this version posted January 25, 2025. The copyright holder for this preprint
(which was not certified by peer review) is the author/funder, who has granted medRxiv a license to display the preprint in perpetuity.
It is made available under a CC-BY 4.0 International license .

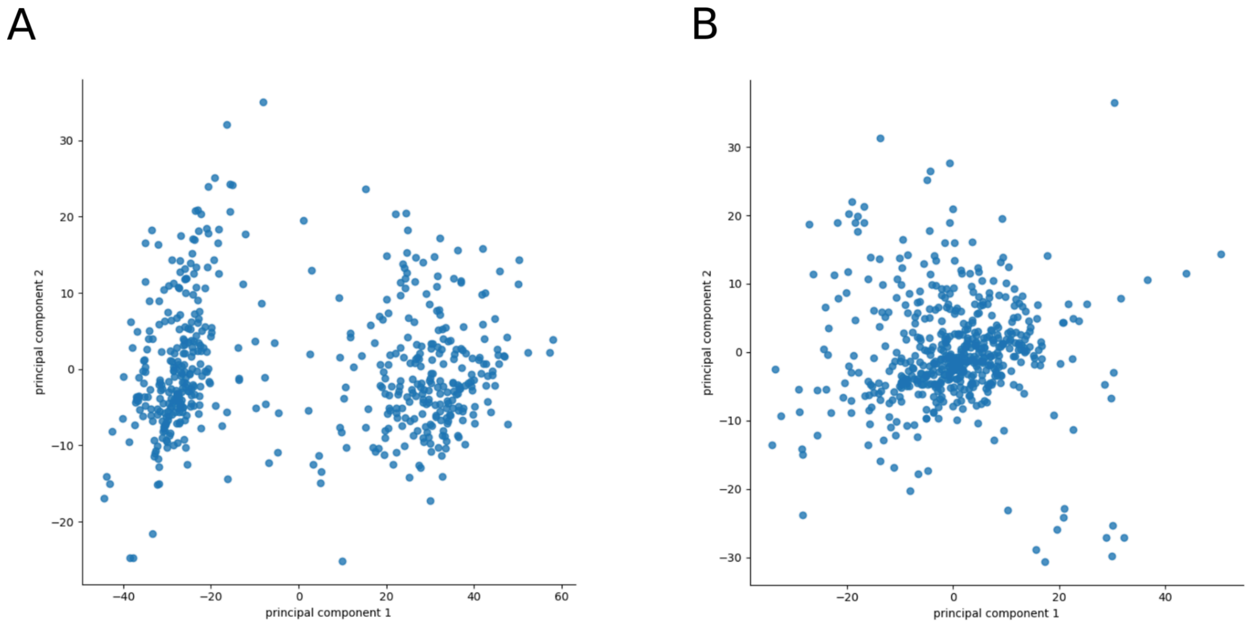

Figure S2: PCA of individual level Olink plasma protein measurements. **(A)** PCA of unadjusted Olink measurements of STARNET samples (n=535). **(B)** PCA of Olink measurements following adjustment for age, sex and the first principal component from the unadjusted data (n=532).

medRxiv preprint doi: https://doi.org/10.1101/2025.01.22.25320723; this version posted January 25, 2025. The copyright holder for this preprint (which was not certified by peer review) is the author/funder, who has granted medRxiv a license to display the preprint in perpetuity.
It is made available under a CC-BY 4.0 International license .

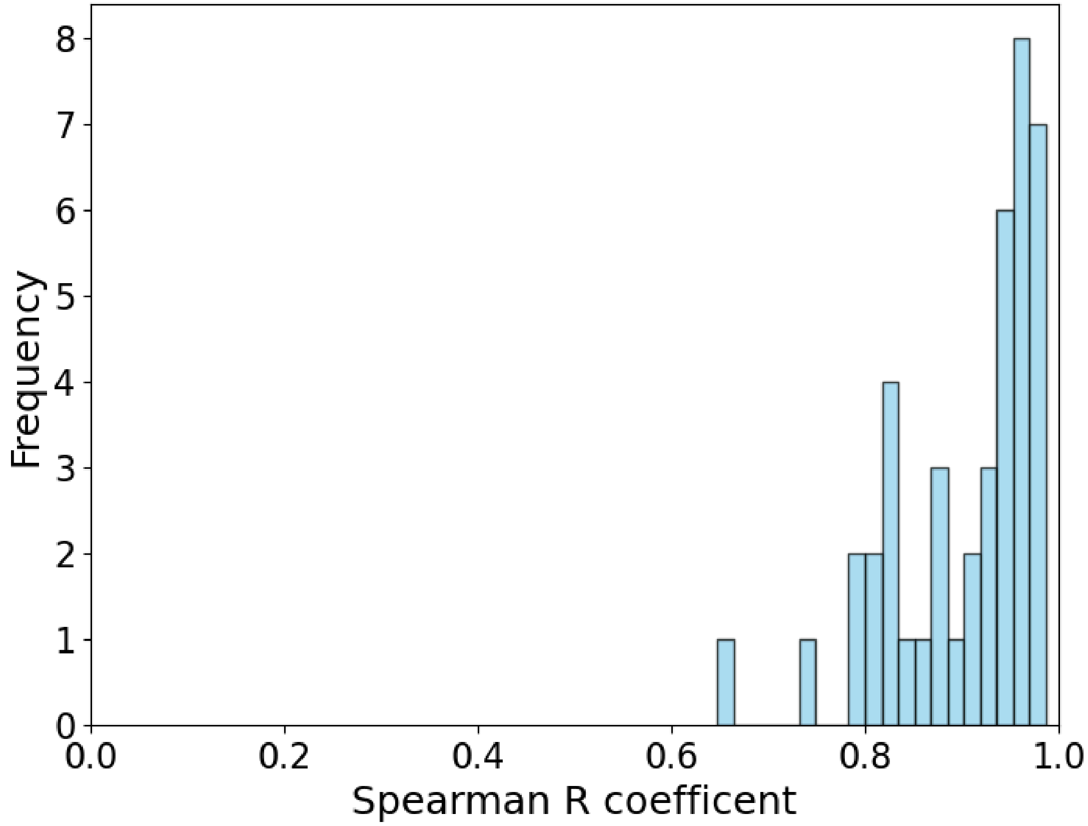

Figure S3: Distribution of pairwise correlations between alternate Olink protein assays for the same protein.
